# Deep RNA sequencing reveals a high frequency of alternative splicing events in the fungus *Trichoderma longibrachiatum*

**DOI:** 10.1186/s12864-015-1251-8

**Published:** 2015-02-06

**Authors:** Bin-Bin Xie, Dan Li, Wei-Ling Shi, Qi-Long Qin, Xiao-Wei Wang, Jin-Cheng Rong, Cai-Yun Sun, Feng Huang, Xi-Ying Zhang, Xiao-Wei Dong, Xiu-Lan Chen, Bai-Cheng Zhou, Yu-Zhong Zhang, Xiao-Yan Song

**Affiliations:** State Key Laboratory of Microbial Technology, Shandong University, Jinan, 250100 China; Marine Biotechnology Research Center, Shandong University, Jinan, 250100 China; Collaborative Innovation Center of Deep Sea Biology, Shandong University, Jinan, 250100 China; Technology Center, Shandong Tobacco Industry Corporation, Jinan, 250013 China

**Keywords:** Alternative splicing, Fungi, RNA-Seq, Intron retention, Transcriptome, *Trichoderma longibrachiatum*

## Abstract

**Background:**

Alternative splicing is crucial for proteome diversity and functional complexity in higher organisms. However, the alternative splicing landscape in fungi is still elusive.

**Results:**

The transcriptome of the filamentous fungus *Trichoderma longibrachiatum* was deep sequenced using Illumina Solexa technology. A total of 14305 splice junctions were discovered. Analyses of alternative splicing events revealed that the number of all alternative splicing events (10034), intron retentions (IR, 9369), alternative 5’ splice sites (A5SS, 167), and alternative 3’ splice sites (A3SS, 302) is 7.3, 7.4, 5.1, and 5.9-fold higher, respectively, than those observed in the fungus *Aspergillus oryzae* using Illumina Solexa technology. This unexpectedly high ratio of alternative splicing suggests that alternative splicing is important to the transcriptome diversity of *T. longibrachiatum*. Alternatively spliced introns had longer lengths, higher GC contents, and lower splice site scores than constitutive introns. Further analysis demonstrated that the isoform relative frequencies were correlated with the splice site scores of the isoforms. Moreover, comparative transcriptomics determined that most enzymes related to glycolysis and the citrate cycle and glyoxylate cycle as well as a few carbohydrate-active enzymes are transcriptionally regulated.

**Conclusions:**

This study, consisting of a comprehensive analysis of the alternative splicing landscape in the filamentous fungus *T. longibrachiatum*, revealed an unexpectedly high ratio of alternative splicing events and provided new insights into transcriptome diversity in fungi.

**Electronic supplementary material:**

The online version of this article (doi:10.1186/s12864-015-1251-8) contains supplementary material, which is available to authorized users.

## Background

Alternative splicing (AS) is crucial for proteome diversity and functional complexity in higher organisms [1]. AS events can be detected by aligning mRNA sequences to the genome sequence, and expressed sequence tags (ESTs) have been widely used in AS detection. For example, using ESTs, the AS ratio has been estimated to be 53% of the multiexonic genes in humans and mice and 19% in fruit flies [2]. The application of high-throughput sequencing technologies to whole transcriptome sequencing (RNA-Seq) prompts our understanding of AS. For example, RNA-Seq revealed that the ratio of multiexonic genes undergoing AS is over 95% in humans [3,4], 42.4% in rice [5], and 61% in *Arabidopsis* [6].

Compared with higher organisms, a much lower AS ratio has been observed in fungi. Based on ESTs, the AS ratio was estimated to be 1.6% in *Magnaporth grisea* [7], 3.6% in *Ustilago maydis* [8] and 4.2% in *Cryptococcus neoformans* [9]. An RNA-Seq study based on the Illumina Solexa platform discovered that 8.6% of the total genes (11.1% of multiexonic genes) are alternatively spliced in the fungus *Aspergillus oryzae* [10]. There are several comparative studies of AS events in multiple species [11-13]. A recent study of 23 fungal species observed that a greater fraction of fungal genes than previously estimated were affected by AS [13]. They estimated that an average of 6.4% annotated genes are affected by AS, and *C. neoformans* has an extraordinary rate of 18% [13]. However, compared with higher organisms, our understanding of AS events in fungi is very limited.

*Trichoderma* spp. are free-living fungi that are highly interactive in root, soil, and foliar environments [14]. They produce a wide range of antibiotic substances, including cell wall degrading enzymes and peptaibols, and parasitize other fungi. Some *Trichoderma* species, e.g., *Trichoderma atroviride* (*Ta*) and *Trichoderma virens* (*Tv*), are used as biocontrol reagents [14]. *Trichoderma reesei* (*Tr*) is the main industrial source of cellulases and hemicellulases [15,16]. The genomes of *Tr*, *Ta*, and *Tv* have been sequenced, providing invaluable resources for the study of *Trichoderma* metabolism. Comparative transcriptomic studies have been performed to gain insights into the metabolism and mycoparasitism of *Trichoderma* spp. [17-22]. However, splice junctions and alternative splicing events in *Trichoderma* have not been studied.

Recently, we sequenced the genome of *Trichoderma longibrachiatum* (*Tl*), which has the smallest genome (~31.7 Mb) among the sequenced *Trichoderma* species [23]. In this study, the *Tl* transcriptome was deep sequenced using Illumina Solexa technology. Comparative transcriptomics for two growth media were performed, and the transcription levels for carbohydrate-active enzymes and other genes were evaluated and compared to gain insights into transcriptional regulation. Furthermore, comprehensive analyses of splice junctions (SJs) and AS events were conducted. This study revealed an unexpectedly high ratio of AS in *Tl*, which is much higher than that observed in the fungus *A. oryzae* and is comparable with those observed in humans and plants. This study provides new insights into the AS landscape in fungi as well as *Trichoderma* physiology.

## Results

### *T. longibrachiatum* transcriptome sequencing

Efforts have been undertaken to discover additional transcribed genes and thus to discover additional SJs and AS events. Initially, *Tl* was cultured in two growth media, the nutrition-rich Potato Dextrose Broth (PDB) and the nutrition-scarce mineral salt medium (MSM). In addition to the mineral salts included in MSM, fungal cell wall, laminarin, cellobiose, and xylan were also included to induce carbohydrate-active enzymes. The fungal cell wall contains chitin and β-1,3 and β-1,6-glucan [24,25] and therefore can induce the expression of chitinases, β-1,3-glucanases, and β-1,6-glucanases. Laminarin, composed of β-1,3- and β-1,6-glucan, can also induce the production of β-1,3-glucanases and β-1,6-glucanases. Cellobiose can induce the expression of cellulases. Xylan can induce the expression of hemicellulases. Secondly, for each medium, the fungal mycelia were collected at 40 hours and at 72 hours, and equal amounts of the 40-hour RNA sample and the 72-hour RNA sample were mixed to prepare a 200-bp paired-end library. Lastly, each 200-bp library was deeply sequenced. The PDB library produced ~1372 Mbp of clean data (~18.5 M clean reads, read length, 73 bp and 75 bp), and the MSM library produced ~1166 Mbp of clean data (~15.8 M clean reads, read length, 73 bp and 75 bp). Collectively, the two libraries produced a total of ~2.5 Gbp of clean data, representing ~80-fold of the *Tl* genome (~31.7 Mb).

**Figure 1:**
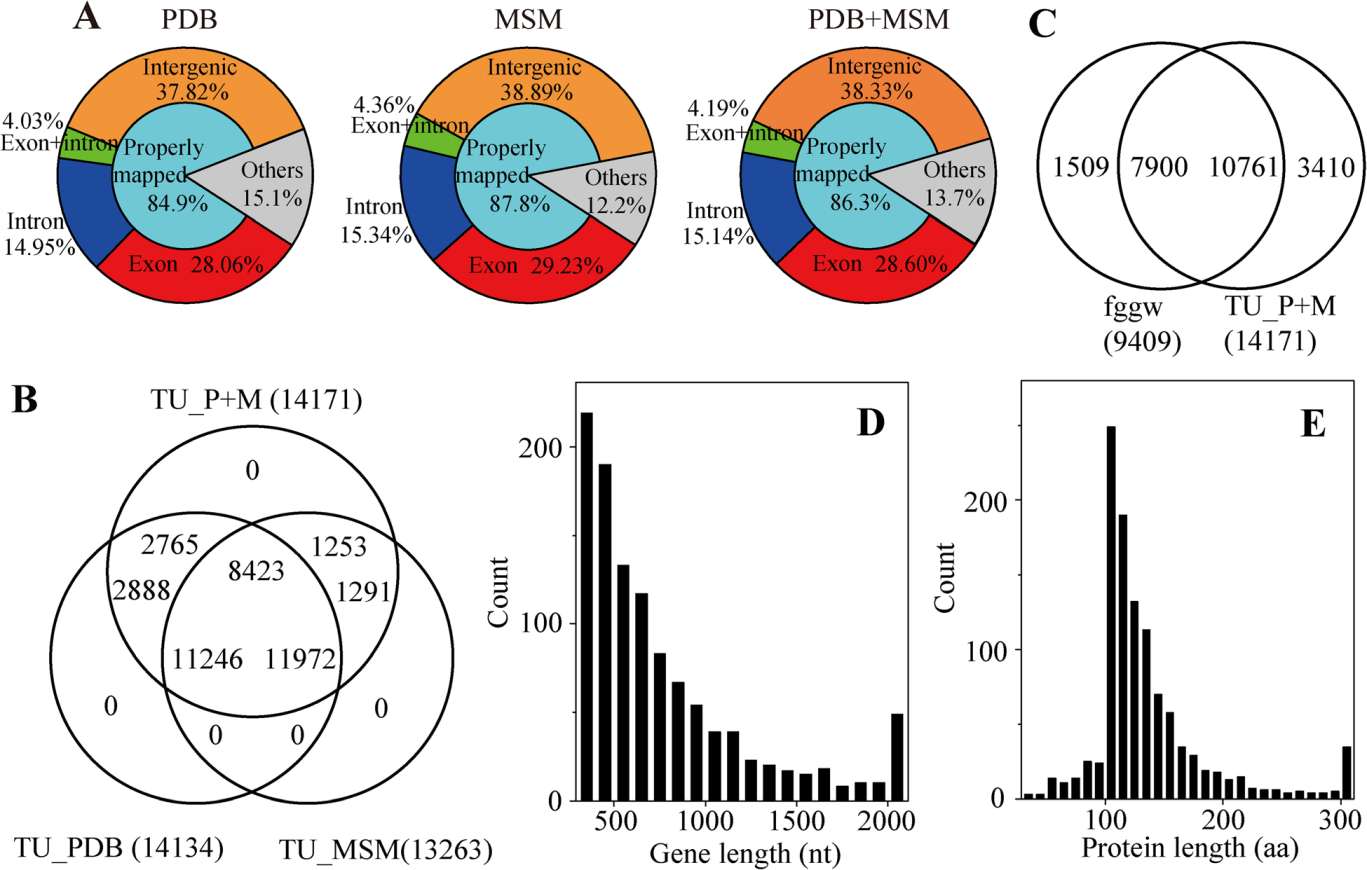
**Statistics of RNA-Seq. (A)** Mapping of RNA-Seq reads to the reference genome. **(B)** Venn diagrams of transcript units (TUs) predicted from PDB library, MSM library, and pooled PDB + MSM libraries. **(C)** Comparison of PDB + MSM TUs with fggw set of gene models. **(D and E)** Length distribution of new gene models **(D)** and new proteins **(E)** predicted based on RNA-Seq.

RNA-Seq reads were mapped to the *Tl* genome using TopHat [26]. As a result, 84.9% of the PDB reads and 87.8% of the MSM reads could be mapped to the *Tl* genome (Figure 1A) with a proper interval between the paired-end reads. The mapped PDB reads were assembled into 14134 PDB transcript units (43.52% of the *Tl* genome). The mapped MSM reads were assembled into 13263 MSM transcript units (38.43% of the *Tl* genome). By pooling the PDB reads and MSM reads, we obtained a total of 14171 PDB + MSM transcript units, which account for over half (52.22%) of the total *Tl* genome (Figure 1B).

### New gene models based on RNA-Seq

In a previous study, a total of 9409 gene models (fggw set) were predicted based on a combination of gene predictors [23]. Comparison of PDB + MSM transcript units with the fggw models revealed 3410 PDB + MSM transcript units that were not included in the fggw models and most likely represent new genes (Figure 1C). All the mapped RNA-Seq reads were assembled into 22231 rnaseq models (see Methods for details). Comparison of the models revealed 1111 protein-coding rnaseq models that did not overlap with the fggw models, tRNA models or rRNA models. The length of these gene models was relatively short (median 607 bp, 121 aa; Figure 1D and E). BLASTP searches revealed that only 21 proteins have close relatives (expect value < 1E-10) in the SwissProt database and 113 proteins have close relatives (expect value < 1E-10) in the NCBI nr database (Additional file 1: Table S1), suggesting that the majority of these newly found RNA-Seq-based gene models represent possible new genes for *Trichoderma*. A previous study revealed that the *Tl* genome encodes 165 glycoside hydrolases, 93 glycosyltransferases, 19 carbohydrate esterases, and four polysaccharide lyases [23]. In this study, annotation of these new RNA-Seq genes revealed two additional glycoside hydrolases, including SMF2RNA_212515 (family 1 glycoside hydrolase) and SMF2RNA_209491 (family 2 glycoside hydrolase).

### Comparison of gene transcription levels in two growth media

**Figure 2:**
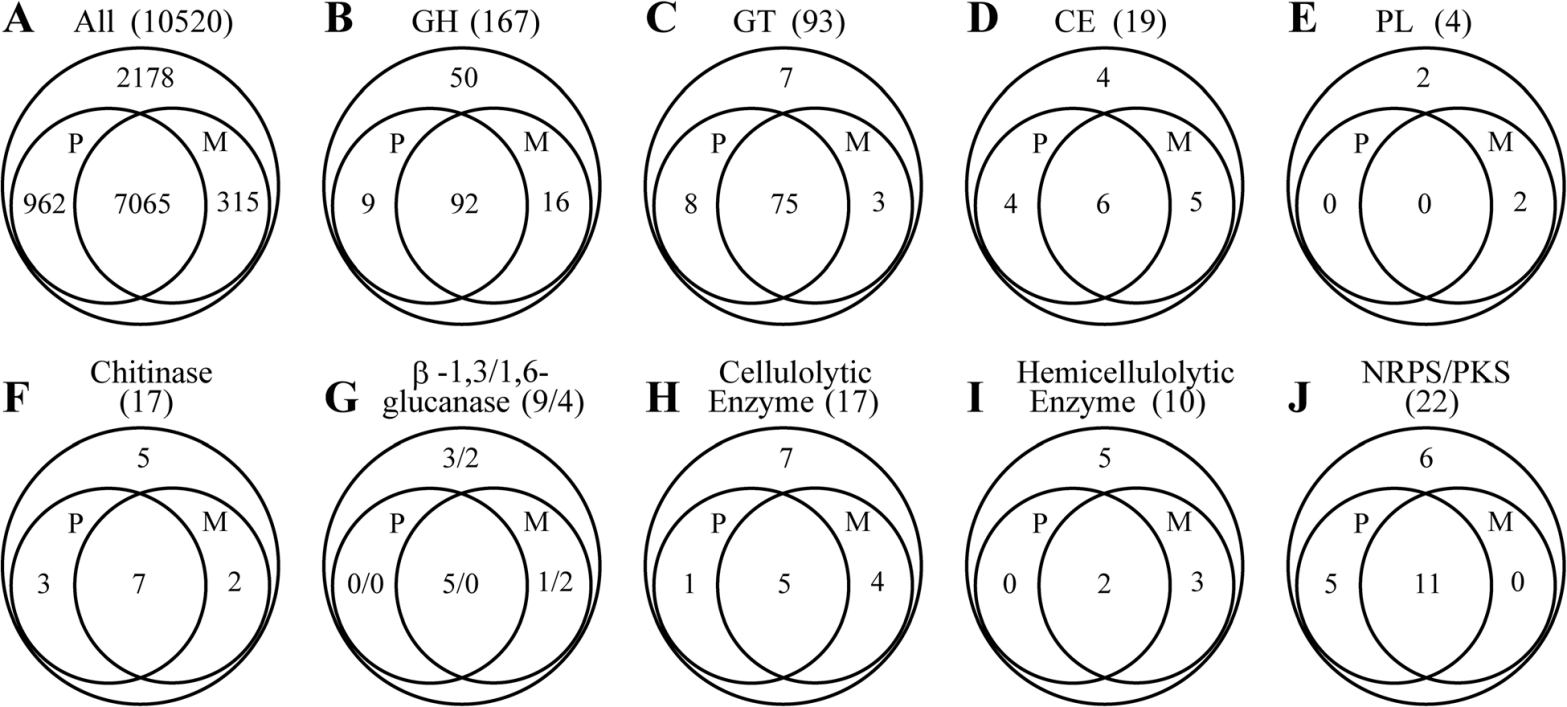
**Comparison of numbers of genes transcribed in different media. (A)** All genes. **(B)** Glycoside hydrolases (GH). **(C)** Glycosyltransferases (GT). **(D)** Carbohydrate esterases (CE). **(E)** Polysaccharide lyases (PL). **(F)** Chitinases. **(G)** β-1,3/1,6-glucanses. Numbers were presented as numbers of β-1,3-glucanses / numbers of β-1,6-glucanses. **(H)** Cellulolytic enzymes. **(I)** Hemicellulolytic enzymes. **(J)** Non-ribosomal peptide synthetases (NRPS) and/or polyketide synthases (PKS). “P” indicates genes transcribed in PDB and “M” indicates genes transcribed in MSM.

The expression level of each gene, measured as the numbers of fragments per kilobase of exon per million fragments mapped, FPKM, was calculated using cufflinks [27]. Comparative analyses revealed large differences in global gene transcription for the two growth media (Figure 2, see Additional file 1: Table S2 for the annotation and expression of all the fggw models and new RNA-Seq models). In PDB, 8027 genes (76.3% of total 10520) were transcribed (from 0.192 FPKM to 1.19E5 FPKM, Figure 2A). Fewer genes (7380, 70.2%) were transcribed in MSM (from 0.336 FPKM to 2.32E5 FPKM, Figure 2A). A total of 7065 genes (67.2%) were transcribed in both media. Among these genes, 974 genes (9.3% of total 10520) had transcription levels increased by 2 to 400-fold in MSM compared with PDB. Additionally, among these genes, 1641 genes (15.6%) had transcription levels that were decreased by 2 to 69.8-fold in MSM compared with PDB.

**Figure 3:**
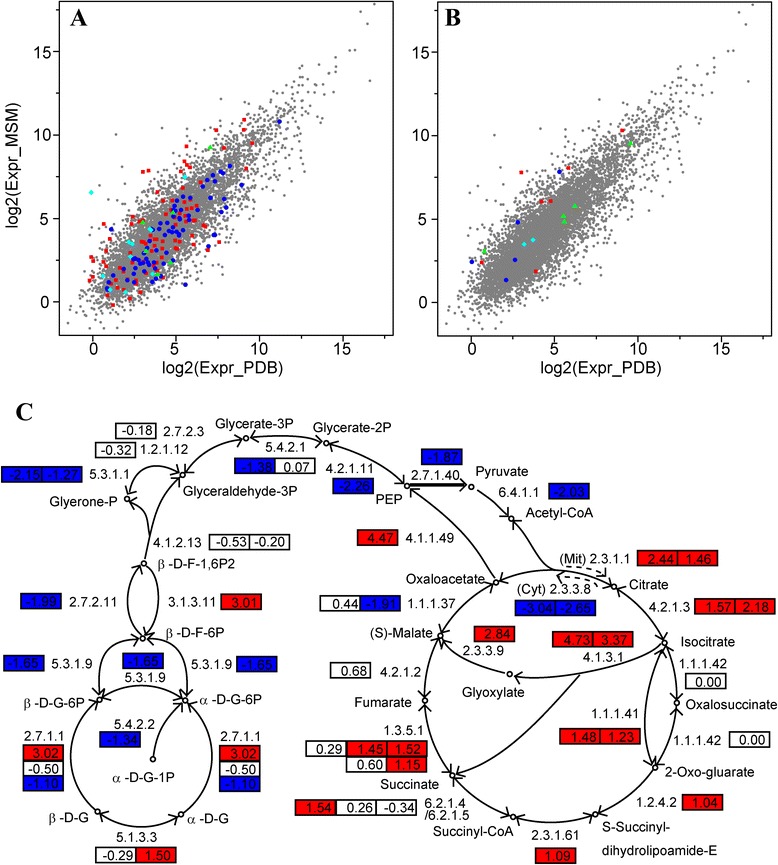
**Comparison of transcription levels of genes transcribed in both media. (A)** Transcription levels of glycoside hydrolases (GH, red squares), glycosyltransferases (GT, blue circles), carbohydrate esterases (CE, green triangles), and non-ribosomal peptide synthetases (NRPS) and/or polyketide synthases (PKS) (cyan diamonds). All the other genes were indicated with grey dots. **(B)** Transcription levels of chitinases (red squares), β-1,3-glucanses (blue circles), cellulolytic enzymes (green triangles), and hemicellulolytic enzymes (cyan diamonds). All the other genes were indicated with grey dots. **(C)** Transcription of genes involved within glycolysis, the citrate cycle and glyoxylate cycle. The numbers in box indicate the log2(fold change) of transcription levels of genes when cultured in MSM compared with in PDB. Positive numbers indicate an increase of transcription levels (numbers ≥1 shown in red) and negative numbers indicate a decrease of transcription levels (numbers ≤ -1 shown in blue) in MSM than in PDB.

Carbohydrate-active enzymes are important in the degradation of biomass and attack of plant fungal pathogens by *Trichoderma* spp. In this study, two growth media were used, and the transcription of all carbohydrate-active enzymes was also analyzed. As shown in Figure 2B, more glycoside hydrolase genes were transcribed in MSM (108) versus PDB (101). Combining both media, 127 glycoside hydrolase genes were transcribed in one or two media (Figure 2B). For those (92) transcribed in both media, 28 increased their transcription over two fold, and 26 decreased their transcription over two fold in MSM compared with PDB (Figure 3A, red squares). For glycosyltransferases, fewer genes (78 versus 83) were transcribed in MSM than PDB (Figure 2C). Combining both media, 86 glycosyltransferase genes were transcribed in one or two media (Figure 2C). For the 75 glycosyltransferase genes transcribed in both media, only two increased their transcription over two fold, but 22 decreased their transcription over two fold (Figure 3A, blue circles). For carbohydrate esterases, ten genes were transcribed in PDB and eleven were transcribed in MSM (Figure 2D, Figure 3A, green triangles). Among the four predicted polysaccharide lyase genes, only two were transcribed in MSM (Figure 2E).

Of the 17 chitinase genes (family 18 glycoside hydrolase) in the *Tl* genome, ten were transcribed in PDB and nine were transcribed in MSM (Figure 2F). Transcription levels varied over a large range (0.805 to 530 FPKM in PDB and 0.538 to 1270 FPKM in MSM, Figure 3B). Transcription of the predominant chitinase, SMF2FGGW_101150, was increased by 2.4-fold (530 FPKM in PDB and 1270 FPKM in MSM). For another six chitinases transcribed in both media, the transcription levels of five (SMF2FGGW_103922, SMF2FGGW_104228, SMF2FGGW_104317, SMF2FGGW_106937, SMF2FGGW_107557, and SMF2FGGW_108810) were up-regulated (by 2.5 to 27.5-fold) in MSM (Figure 3B, red squares).

For the nine β-1,3-glucanases, five were transcribed in PDB, and one additional enzyme was transcribed in MSM at 0.998 FPKM (Figure 2G). The predominant β-1,3-glucanase, SMF2FGGW_100213, has a 5.8-fold increase in the transcription level (from 38.8 to 226 FPKM). For another four β-1,3-glucanases transcribed in both media, two had elevated transcription levels in MSM (Figure 3B, blue circles). The *Tl* genome encodes four β-1,6-glucanases; however, none of these β-1,6-glucanases were transcribed in PDB. SMF2FGGW_104549 and SMF2FGGW_108952 were transcribed in MSM (Figure 2G).

Among the 17 cellulolytic enzymes in the *Tr* genome, only six were transcribed in PDB and nine were transcribed in MSM (Figure 2H). The predominant enzyme is SMF2FGGW_104689 (GH12). It has almost the same transcription level in the two media (757 FPKM in PDB and 732 FPKM in MSM), suggesting that this enzyme is not induced by cellobiose. Among the four enzymes that were transcribed in both media (Figure 3B, green triangles), only one enzyme had elevated transcription in MSM (SMF2FGGW_101278, from 1.74 FPKM in PDB to 8.31 FPKM in MSM).

The *Tr* genome encodes ten hemicellulolytic enzymes. Only two were transcribed in PDB (SMF2FGGW_105688 and SMF2FGGW_106057, Figure 2I). In the induction medium MSM, the transcription of these two enzymes was remained almost unchanged (Figure 3B, cyan diamonds); however, an additional three enzymes were transcribed (SMF2FGGW_100507, SMF2FGGW_101883, and SMF2FGGW_103677).

The transcription of most enzymes related to starch metabolism, glycolysis/gluconeogenesis, citrate cycle, and glyoxylate cycle were altered in MSM compared with PDB (see in Additional file 1: Table S2 for the transcription levels for all genes). In MSM, the transcription of genes related to starch metabolism was down-regulated (Figure 3C), which is in agreement with the lack of starch in MSM. The transcription of most genes involved in glycolysis was down-regulated, and the transcription of the gluconeogenesis-related enzyme, fructose-1,6-bisphosphatase (EC:3.1.3.11, SMF2FGGW_3061), was up-regulated (Figure 3C). The majority of genes related to the citrate cycle and glyoxylate cycle were up-regulated (Figure 3C), suggesting that energy generation in MSM was up-regulated at the transcriptional level. The genome annotation revealed six genes encoding catalases (EC:1.11.1.6). Further analysis revealed that the transcription of two catalases (SMF2FGGW_4095 and SMF2FGGW_8095) was decreased (with statistical significance), which also indicates low metabolism and energy levels in the nutrient-scarce MSM.

For the 22 annotated NRPS-PKS genes in the genome, 16 were transcribed in PDB and eleven were transcribed in MSM (Figure 2J). However, for the eleven genes transcribed in both media, the transcription of four genes was up-regulated over two fold, while no genes had down-regulated transcription over two fold (Figure 3A, cyan diamonds; Additional file 1: Table S2). The up-regulated genes include the longest gene in the genome (SMF2FGGW_105489, 5.18 to 11.0 FPKM), which is responsible for the synthesis of 20-aa peptaibols, and a conidial yellow pigment biosynthesis polyketide synthase (SMF2FGGW_103752, 45.8 to 179 FPKM). The transcription of the second longest gene in the genome (SMF2FGGW_101095), which is responsible for the synthesis of 12-aa peptaibols, was also increased (11.2 to 19.8 FPKM), although by a slightly lower amount.

### Splice junctions

**Figure 4:**
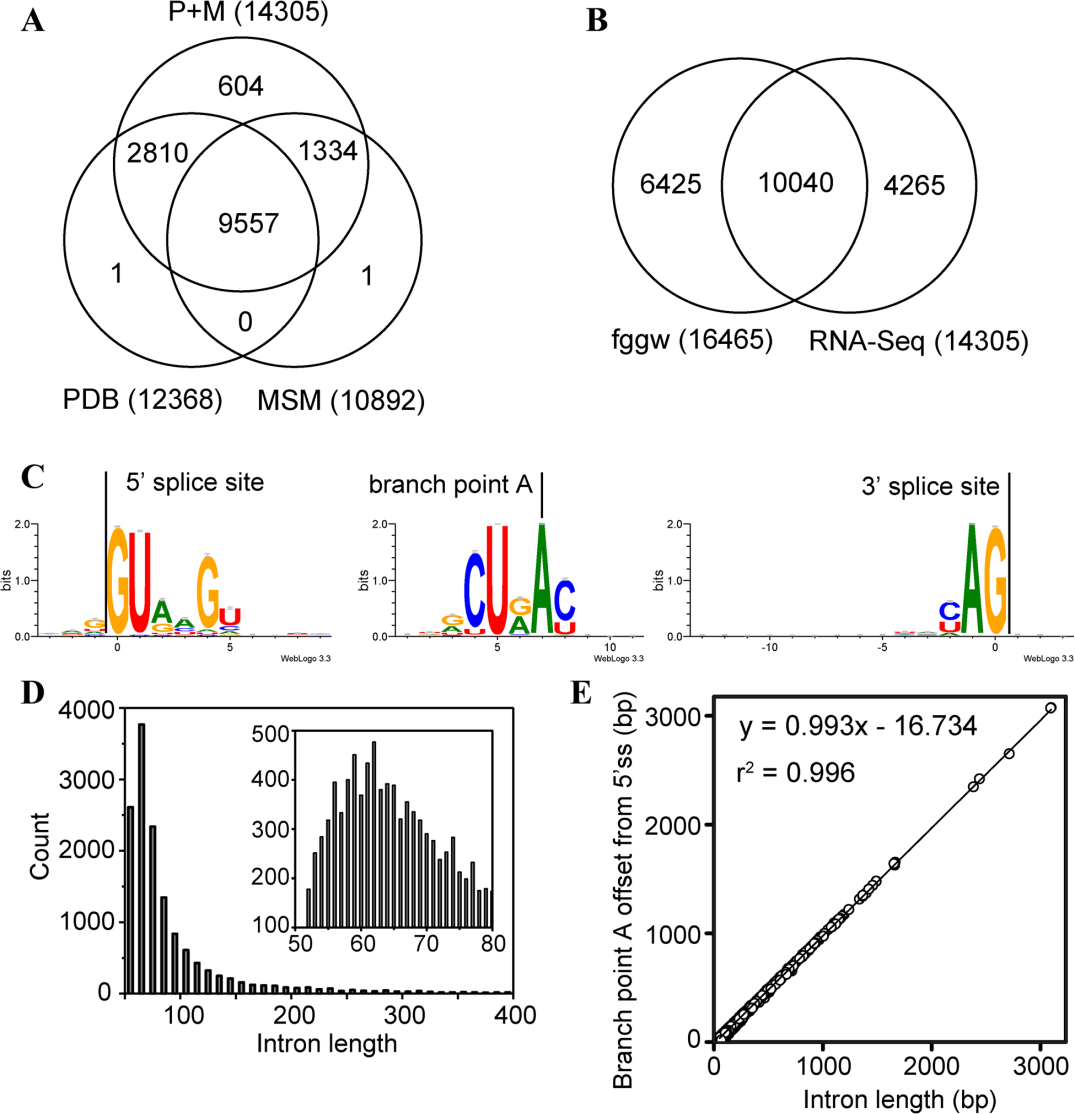
**Characteristics of predicted splicing junctions (SJs). (A)** Venn diagrams of SJs predicted based on PDB library, MSM library, and pooled PDB + MSM libraries. **(B)** Venn diagrams of SJs based on the fggw set of gene models and those based on RNA-Seq. **(C)** Sequence logos of 5’ splice site (left), branch site (middle), and 3’ splice site (right) sequence of introns. Logos were created using WebLogo 3 [30]. **(D)** Distribution of lengths of introns based on RNA-Seq. **(E)** The relation between the branch point A offset from 5’ splice site and the intron length.

SJs were predicted using TopHat [26], with a subsequent filtering and correction process (see Methods for details). By examining the read alignment file (“accepted_hits.sam”) reported by TopHat, we obtained a set of 14678 SJs that were supported by at least two uniquely mapped paired-end reads from pooled PDB and MSM reads. By searching the intron sequence using CURAY (where R is purine and Y is pyrimidine) as the primary motif, UURAY as the secondary motif, and a modified YURAY as the third motif (See Methods), we located the branch site for almost all of the SJs (14642 of total 14678). The distance from the branch point A to 3’ss was 18.5 ± 22.3 bp (median 15 bp). We only included 14394 SJs that had a branch point A that was within 60 bp of 3’ss in the following analysis. In addition, we performed an SJ correction by excluding 88 SJs that partially overlapped neighboring SJs (located within 10 bp) of the same SJ length. We also excluded one SJ that had a sequence that contained an ‘N’. The obtained 14305 SJs were referred to as the TopHat_Rev version and were used for the following analyses (see Additional file 1: Table S3 for all SJs). Further analysis of the PDB and MSM samples suggested that only 9557 SJs (66.8% of total 14305) were included in both the PDB and MSM samples (Figure 4A). The fggw set models included a total of 16465 introns. Comparison of RNA-Seq SJs with the introns in fggw models revealed that the RNA-Seq uncovered 4265 new SJs that were not included in the fggw models (Figure 4B).

Among the 14305 SJs, 13993 (97.8%) had the canonical splice site pair GT-AG, 243 (1.7%) had the noncanonical splice site pair GC-AG, and 69 (0.5%) had the noncanonical splice site pair AT-AC. The 5’ss sequences and 3’ss sequences are shown in Figure 4C (left and right). The length of RNA-Seq introns was 102.3 ± 112.2 bp (median 72 bp, with a peak at approximately 55 bp - 65 bp) (Figure 4D). The branch site sequence is shown in Figure 4C (middle). The offset of branch point A from 3’ss was approximately 16.5 ± 7.0 bp (median 15 bp, with a peak at 11 bp - 14 bp), which is similar to *Schizosaccharomyces pombe* (13 bp), *Aspergillus nidulans* (15 bp), *Neurospora crassa* (20 bp), and *C. neoformans* (18 bp) and shorter than *Saccharomyces cerevisiae* (36 bp) [28]. The offset from the 5’ss was correlated with intron length (Figure 4E). The alignment of 3’ss sequences did not reveal the polypyrimidine tract (Figure 4C, right). A search for six consecutive nonadenine nucleotides containing at least three uridines in the intron sequence revealed that 12332 introns (86.2% of total 14305) have polypyrimidine tracts. Different from humans and plants, most introns (9871, 69.0% of 14305) had their polypyrimidine tract between the 5’ss and branch site. Similarly, a large ratio of the majority of introns in *S. cerevisiae* (28.5%), *S. pombe* (62.1%), *A. nidulans* (52.8%), *N. crassa* (47.5%), and *C. neoformans* (47.9%) have polypyrimidine tracts in the region between the 5’ ss and branch site [28].

### High frequency of alternative splicing events in *Trichoderma*

**Figure 5:**
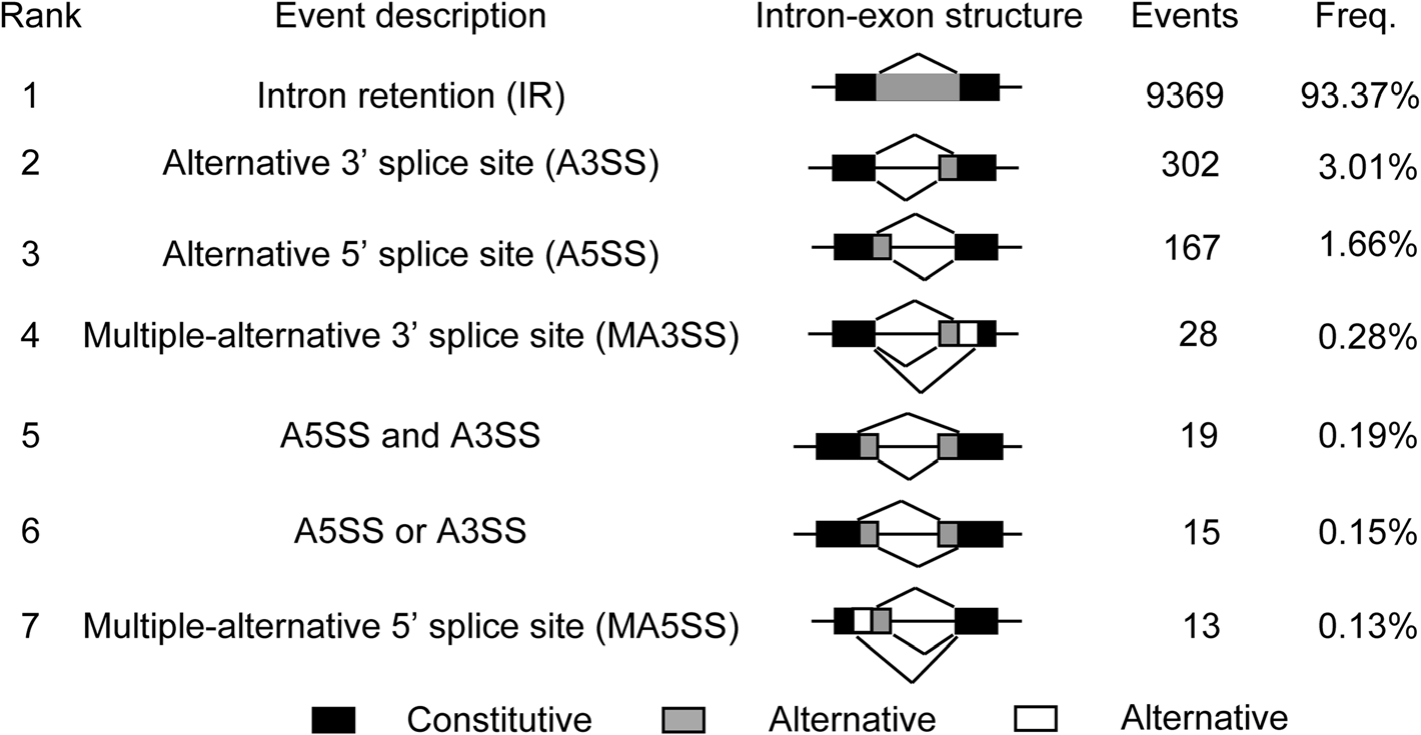
**Schematic diagrams of major types of alternative splicing events detected in** ***T. longibrachiatum*** **transcriptome.**

We performed a computational analysis of the most common AS events, including intron retention (IR), alternative 5’ splice site (A5SS), alternative 3’ splice site (A3SS), and exon skipping, in *Tl* (see Methods for details). By comparing the coordinates of all the RNA-Seq SJs (TopHat_Rev), we obtained 13174 clusters that were used for the identification of AS events. A total of 10034 AS events were obtained using our procedure. The major types of AS events detected in *Tl* are shown in Figure 5. The details of all SJ clusters are presented in Additional file 1: Table S4.

For the 12509 clusters that each contained only one SJ, 9369 were identified as IR and 3140 were identified as constitutive introns (IC). For the 503 clusters that each contained two SJs, 167 were identified as A5SS and 302 as A3SS. In addition to A5SS and A3SS, we also identified two other types of AS events. As shown in Figure 5, the two SJs share a portion of intronic regions but do not share a 5’ splicing site (5’ss) or a 3’ splice site (3’ss). Following Marquez et al. [8], we denoted them as “A5SS and A3SS” events (19 cases) and “A5SS or A3SS” events (15 cases).

In addition to clusters containing one or two SJs, we also identified 162 clusters that each contained three or more SJs. No exon-skipping events were identified in these clusters. These clusters can be regarded as different combinations of the above basic AS events. A simple example is that all SJs in the cluster share the same 5’ss or 3’ss and these events were denoted as a multiple-alternative 5’ splicing site (MA5SS) and a multiple-alternative 3’ splicing site (MA3SS), respectively. As shown in Figure 3, 28 MA3SS, including 25 MA3SS(3) and 3 MA3SS(4), and 13 MA5SS, including 12 MA5SS(3) and 1 MA5SS(5), were observed, where the numbers in parentheses indicate the total number of SJs in the cluster. In addition to MA5SS and MA3SS, there were a total of 121 complicated AS events that are difficult to classify. We encoded these clusters by combining the total number of SJs in the cluster and the numbers of A5SS, A3SS, “A5SS and A3SS”, and “A5SS or A3SS” identified by a pairwise comparison of all SJs in the cluster.

Therefore, the above analysis revealed an unexpected high ratio of AS (76.2%, 10034 AS events out of total 13174 SJ clusters) in *Tl*. Compared with those observed in *A. oryzae* [10], the number of total AS events (10034 versus 1375), IR (9369 versus 1259), A5SS (167 versus 33), and A3SS (302 versus 51) are approximately 7.3, 7.4, 5.1, and 5.9-fold higher. Mapping SJs to the fggw models revealed that the alternatively spliced SJs could be mapped to 4601 fggw models (48.9% of total fggw models), among which 4486 were multiexonic genes and 115 were monoexonic genes. Therefore, approximately 48.9% of genes (63.2% of multiexonic fggw models) in *Tl* are alternatively spliced. This ratio of alternatively spliced genes was much higher than what has been observed in other fungi, including 1.6% (134) in *M. grisea* [7], 3.6% (224) in *U. maydis* [8], and 8.55% (1032) in *A. oryzae* [10].

To validate the predicted AS events, 45 IR, three A5SS, and five A3SS events were randomly selected and verified using RT-PCR (Additional file 2: Figures S1 and S2 and Additional file 1: Table S5). For each selected AS event, a pair of exclusive PCR primers were designed and the PCR products were sequenced. As a result, 31 IR (69% of 45 selected IR), two A5SS (67% of three selected A5SS), and five A3SS (100% of five selected A3SS) events were validated. These results suggested that the predicted AS events represent true AS events.

### Length, GC content, and splice site scores of alternatively spliced introns

**Figure 6:**
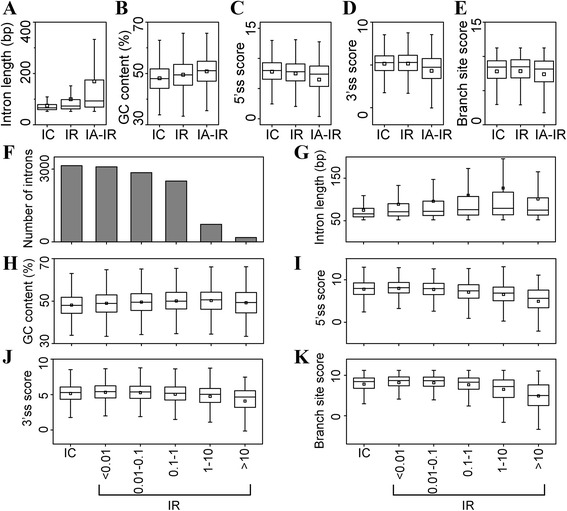
**Features of alternative spliced introns. (A-E)** Length **(A)**, GC content **(B)**, 5’ss score **(C)**, 3’ss score **(D)**, and branching site score **(E)** distribution of different types of introns. IC, constitutive introns; IR, retained introns; IA-IR, alternatively spliced introns except retained introns. **(F)** Number of constitutive introns (IC) and retained introns (IR) in each intron retention ratio bin. **(G-K)** Length **(G)**, GC content **(H)**, 5’ss score (IC), 3’ss score **(J)**, and branching site score **(K)** distribution of constitutive introns (IC) and retained introns (IR) in each intron retention ratio bin. Scores of 5’ss, 3’ss, and branching site sequences were calculated according Sheth et al. [36] and raw scores are presented here.

All of the types of introns were carefully studied and compared (see Additional file 1: Table S6 for details of each type of intron; see Additional file 2: Figure S3 for the sequence logo for each type of intron; see Additional file 1: Table S7 for details of statistical analyses performed in this study). We compared the length of different types of introns and observed that the length of retained introns (IR, 99.7 ± 96.3 bp, median 73 bp) was longer than constitutive introns (IC, 74.7 ± 30.0 bp, median 66 bp; Mann-Whitney-Wilcoxon test, P = 4.90 × 10^−76^) and the length of alternatively spliced introns except retained introns (IA-IR, 169.3 ± 218.8 bp, median 93 bp) is longer than retained introns (P = 4.03 × 10^−84^; Figure 6A). This result suggests that intron length can be used as a parameter to distinguish constitutive introns, retained introns, and alternatively spliced introns except retained introns. Similarly, GC content can also be used as a parameter to distinguish constitutive introns, retained introns, and alternatively spliced introns except retained introns. The comparison of GC content revealed that constitutive introns have the lowest GC content (48.1 ± 5.7, median 48.0), retained introns (49.5 ± 5.9, median 49.5) have a higher GC content (IR > IC, P = 4.48 × 10^−34^), and alternatively spliced introns except retained introns (50.8 ± 5.8, median 51.1) have the highest GC content (IA-IR > IR, P = 2.61 × 10^−17^; Figure 6B). Further analysis of the 5’ss scores demonstrated that retained introns had lower 5’ss scores than constitutive introns (P = 4.90 × 10^−8^) and alternatively spliced introns except retained introns had lower 5’ss scores than retained introns (P = 0; Figure 6C). Therefore, alternatively spliced introns had weaker 5’ss signals than constitutive introns, and the 5’ss signals of retained introns are between those of constitutive introns and alternatively spliced introns except retained introns. The order of 3’ss scores and branch site scores were different from 5’ss scores. Constitutive introns had similar 3’ss scores to retained introns (IC < IR, P = 0.121; Figure 6D) and lower branch site scores than retained introns (P = 0.0240; Figure 6E), while retained introns had both higher 3’ss scores and higher branch site scores than alternatively spliced introns except retained introns (3’ss, P = 0; branch site, P = 5.06 × 10^−10^; Figures 6D-E).

We calculated the intron retention ratio (IRR) of each retained intron and grouped retained introns into bins based on IRR. The results of this analysis determined that a large number (5954, 63.6% of total 9369 retained introns) of retained introns had an IRR lower than 0.1, among which 3097 (33.1%) retained introns had an IRR lower than 0.01 (Figure 6F). Further comparison of constitutive introns with different bins of retained introns determined that all of the bins of retained introns had longer lengths than constitutive introns (P ≤ 3.86 × 10^−9^, Figure 6G) and a higher GC content than constitutive introns (P ≤ 0.00966, Figure 6F). This result further supported the above result that retained introns have longer lengths and higher GC contents than constitutive introns. In addition, retained intron bins with a higher IRR generally had longer lengths (Figure 6G) and higher GC contents (Figure 6H). Similarly, retained intron bins with a higher IRR had lower 5’ss scores, 3’ss scores, and branch site scores (Figures 6I-K). Unexpectedly, the scores of constitutive introns were not higher than all of the retained intron bins, rather, they were nearer to the retained intron bin [0.01, 0.1) or between bins [0.01, 0.1) and [0.1, 1) (Figures 6G-I).

In summary, retained introns, alternatively spliced introns except retained introns, and constitutive introns have different lengths, GC contents, and splice site scores. Retained introns are between constitutive introns and alternatively spliced introns except retained introns. Retained introns with a low IRR are more similar to constitutive introns than those with high IRRs.

### The relative frequencies of A5SS and A3SS isoforms

**Figure 7:**
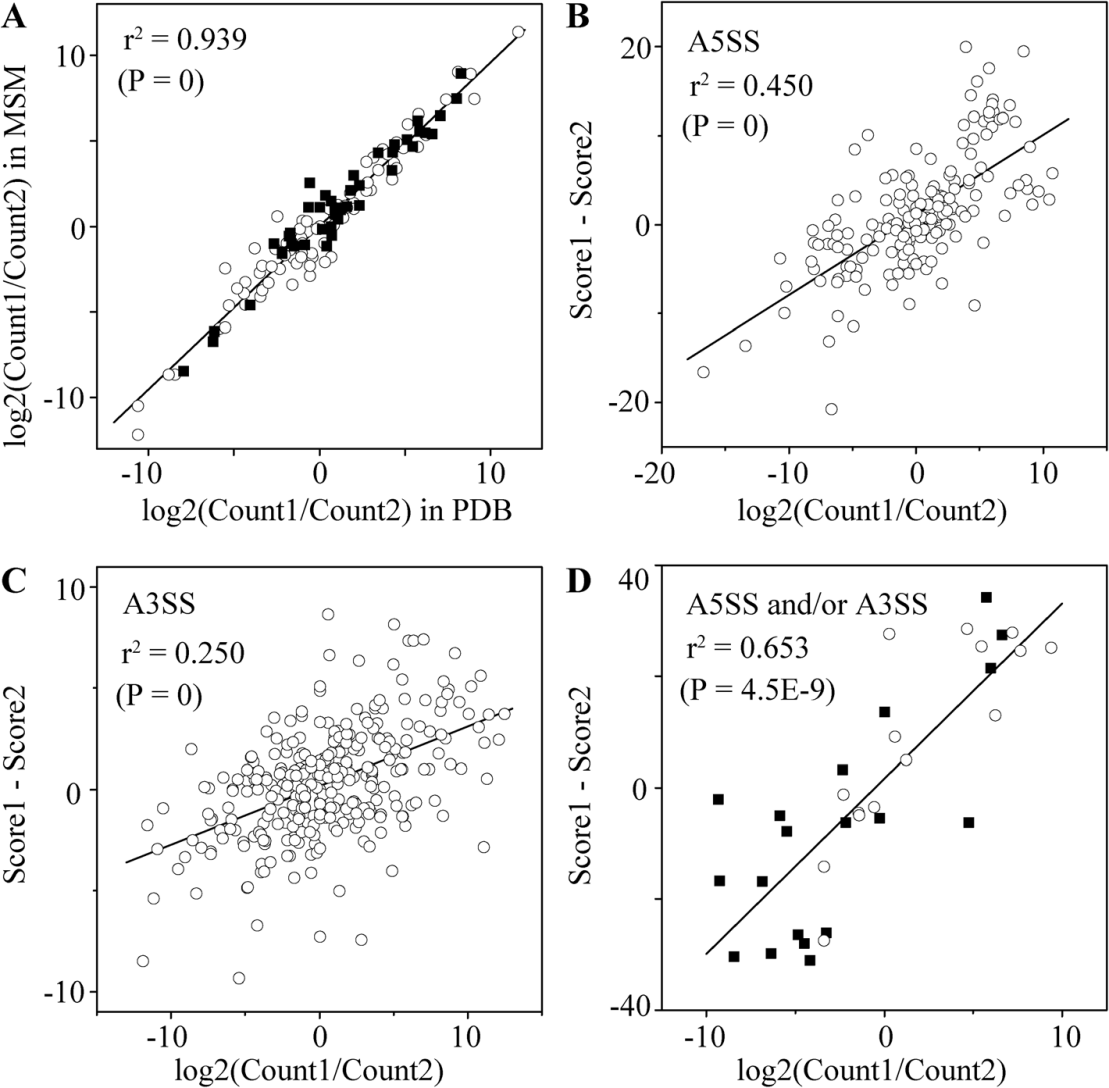
**The relation between splice site scores and isoform relative frequencies. (A)** The relation between isoform relative frequencies in PDB and that in MSM. Filled squares, A5SS; open circles, A3SS. **(B)** The relation between the difference in 5’ss scores and the relative frequencies of A5SS isoforms. **(C)** The relation between the difference in 3’ss scores and the relative frequencies of A3SS isoforms. **(D)** The relation between the difference in the sum of 5’ss and 3’ss scores and the relative frequencies of “A5SS and A3SS” (filled squares) and “A5SS or A3SS” (open circles) isoforms.

We analyzed the isoform frequencies of A5SS and A3SS in PDB and MSM and observed that the relative frequencies of isoforms in PDB were similar to MSM (Figure 7A). This result suggests that the relative frequencies of A5SS and A3SS isoforms are not determined by the external factors from culture media. The major difference between the isoforms was that they had different 5’ss sequences in A5SS or different 3’ss sequences in A3SS. Therefore, the relative frequency of isoforms may be affected by the 5’ss and 3’ss sequences. We examined the relationship between the 5’ss/3’ss sequence and the relative frequency of the isoforms. As shown in Figure 7B, the difference in isoform 5’ss scores was correlated with the logarithm of the isoform frequency ratio (r^2^ = 0.450, P = 0). The difference in isoform 3’ss scores for A3SS was also correlated with the logarithm of the isoform frequency ratio, although the correlation coefficient was low (r^2^ = 0.250, P = 0, Figure 7C). In addition, we also examined the isoforms of “A5SS and A3SS” and “A5SS or A3SS” and observed that the difference in the sum of 5’ss scores and 3’ss scores was well correlated with logarithm of the isoform frequency ratio (r^2^ = 0.653, P = 4.5 × 10^−9^, Figure 7D). These results suggest that 5’ss and 3’ss sequences affect the relative frequencies of A5SS and A3SS isoforms.

## Discussion

The comparative transcriptomics of *Tl* cultured in control medium, PDB, and induction medium, MSM, revealed an abundance of chitinases, β-1,3/1,6-glucanses, cellulolytic enzymes, and hemicellulolytic enzymes that are most likely transcriptionally regulated in response to extracellular polysaccharides. However, although enzymes of different classes may hydrolyze different substrates or different types of linkages in complex substrates, cross talk between the transcriptional regulation of different enzymes cannot be excluded, i.e., the transcription level of one enzyme may also be affected by the other.

In control medium PDB, dextrose and starch are the main carbon sources. In induction medium MSM, polysaccharides, including chitin, β-1,3/1,6-glucan, cellobiose, and xylan, are the main carbon sources for *Tl*. Compared with dextrose and starch, these polysaccharides are more difficult to utilize; therefore, it is expected that *Tl* suffers starvation when grown in MSM. The transcriptional down-regulation of glycolysis-related genes and up-regulation of gluconeogenesis-related genes are inconsistent with the low concentration of glucose in MSM. Therefore, the transcriptional up-regulation of citrate cycle and glyoxylate cycle-related genes may be used to compensate the low energy level of *Tl* cells.

The observed frequency of AS varies among kingdoms. Compared with higher organisms, fungi exhibit less frequent AS events. Early studies based on ESTs determined that 1.6% of genes in *M. grisea* [7], 3.6% of genes in *U. maydis* [8] and 4.2% of genes in *C. neoformans* [9] are alternatively spliced. A comparative study based on ESTs also indicated a low AS frequency (0 - 5%) for fungi *S. cerevisiae*, *S. pombe*, *Encephalitozoon cuniculi*, and *C. neoformans* [11]. As more EST data became available, more AS events were observed in fungi. In a recent study examining 23 fungi, the AS ratio was estimated at 7.9% for *M. grisea* and 18% for *C. neoformans* [13]. The extraordinary AS rate (18%) for *C. neoformans* represents the highest AS rate reported for fungi.

In addition to ESTs, RNA-Seq has also been used in AS analyses. An RNA-Seq study based on the Illumina Solexa platform revealed that 8.55% of genes (11.1% of multiexonic genes) produce two or more AS isoforms in the fungus *A. oryzae* [10]. This AS ratio is similar to the ratio reported for *A. nidulans* (7.3%) and *A. niger* (9.5%) in a comparative study of 23 fungi [13]. In this study, we performed whole transcriptome sequencing of *Tl* using the Illumina Solexa platform. A comprehensive analysis revealed that 48.9% of genes are alternatively spliced. This ratio is not only much higher than fungus *A. oryzae* (8.55%) [10] and closely related *T. reesei* (2.5%) [13], but is also comparable to that in rice (42.4% of multiexonic genes) [5] and *Arabidopsis* (61% of multiexonic genes) [6]. The AS ratio difference observed between *Tl* and *A. oryzae* is likely a result of transcriptome differences because both studies performed the whole transcriptome sequencing using the Illumina Solexa platform.

Splice recognition appears to be different in different kingdoms. A cross-kingdom analysis suggested that the exon definition mechanism is most common in multicellular animals, while the intron definition mechanism predominates in fungi and most protists [12]. Intron retention is the major type (93.4%) of AS events in *Tl*. This high ratio of intron retention suggests that the intron definition mechanism most likely also predominates in *Tl*. Although this ratio is similar to the fungus *A. oryzae* (91.56%) [10], the number of retained introns (9369) is 7.3-fold higher than the fungus *A. oryzae* (1259) [10]. The number of retained introns may be overestimated because of possible DNA contamination and the presence of (immature) pre-mRNA. In this study, DNA contamination can be excluded by using selective RNA isolation. IRR analysis determined that 63.6% of total retained introns have lower IRRs (<0.1). It is unclear whether these rare IR events (especially for IRR < 0.01) have effects in cells. If these retained introns (IRR < 0.1, 5954 in number) were excluded from our dataset, the number of retained introns would be 3415, and the total number of AS events would be 4080, both still exceeding those in *A. oryzae* (1259 retained introns and 1375 total AS) [10]. In this case, the ratio of alternatively spliced introns would also be very high (~31.0%).

Compared with constitutive introns, retained introns have longer lengths, higher GC contents, and lower splice site scores. In contrast, there is no significant difference in intron length between retained introns and constitutive introns in *Arabidopsis* [6]. In addition, the difference in intron length and GC content between retained introns and constitutive introns implies that the predicted retained introns in this study are most likely valid, rather than from genomic DNA contamination or (immature) pre-mRNA. Analyses determined that the splice site sequences of alternatively spliced introns (except retained introns with low IRRs) tend to have lower scores and be less conserved than constitutive introns (Additional file 2: Figure S1). It is possible that the low conservation of splice site sequences (as well as other factors, e.g., intron length, GC content, etc.) prompts the emergence of AS, while the resultant AS subsequently reduces the conservation of the splice site sequence.

The criteria for identification affect the number of predicted AS. In this study, an intron was classified as an IR when it had at least one intronic site supported by one or more reads. We also applied a more stringent criterion to define an IR, that is, an intron was classified as an IR when all the intronic sites were supported by at least two independent reads. Of the 9369 predicted IR, 2710 met this stringent criterion and were referred to as IR (high coverage), and the other IR were referred to as IR (low coverage). The sequence features for both IR (high coverage) and IR (low coverage) are presented in Additional file 2: Figure S4. When only these IR (high coverage) were considered, the total number of AS are 3375 and the AS ratio is 18.9% (1776 fggw models). This ratio is much lower than 48.9%, but is still similar to the highest reported AS ratio for fungi (~18% for *C. neoformans*). In addition, the length, GC content, splice site scores were also analyzed for each IRR bin of IR (low coverage). It was observed that all IRR bins of IR (low coverage) have a longer length and higher GC content compared with IC, and high IRR bins have lower 5’ss scores, 3’ss scores and branch site scores compared with IC (Additional file 1: Table S7 and Additional file 2: Figure S4), suggesting that these IR (low coverage) may represent true IR.

## Conclusions

In summary, this study deep sequenced the transcriptome of filamentous fungus *T. longibrachiatum*. A comprehensive analysis of AS events revealed that 76.2% of splicing sites were alternatively spliced. The ratio of alternatively spliced genes in *T. longibrachiatum* is up to 48.9%, much higher than reported for *Aspergillus oryzae* (8.55%), suggesting that AS may be important for transcriptome diversity in *T. longibrachiatum*. Alternatively spliced introns have different sequence features, including longer intron lengths, higher GC contents, and lower-scored splice site sequences. Our analysis demonstrated a correlation between these factors and AS, although it is uncertain to what extent these factors affect AS. In addition, our analysis also indicated a correlation between splice site sequence and the relative frequencies of the isoforms in A5SS and A3SS, suggesting that splice site sequence affects the relative frequencies of isoforms in A5SS and A3SS. Collectively, this study provides new insights into the AS landscape in fungi.

## Methods

### Fungal strain and cultivation conditions

*T. longibrachiatum* SMF2 was maintained on potato dextrose agar (Sigma) plates. For genome and transcriptome sequencing, strain SMF2 was grown in two liquid media, PDB and MSM, with shaking at 120 rotations per minute for 72 hours at 28°C. PDB contained 0.2% PDB (Sigma) and 0.2% yeast extract (Sigma). MSM contained 0.6% KH_2_PO_4_, 0.6% (NH_4_)_2_SO_4_, 0.25% MgSO_4_∙7H_2_O, 0.25% CaCl_2_, 0.2% yeast extract (Sigma), 0.2% cell wall of a pathogenic *Fusarium* strain, 0.2% laminarin (Sigma), 0.2% cellobiose (Sigma), and 0.5% xylan (Sigma). Fungal cell walls were extracted by centrifugation (4000 × g, 20 min) after ultrasonication.

### Transcriptome sequencing

For transcriptome sequencing, fungal mycelia were collected at 40 hours and 72 hours for each medium and were preserved in TRIzol® Reagent (Invitrogen). Extraction of total RNA, poly(A)-selection of mRNA, cDNA library construction, and Illumina Solexa sequencing were performed at BGI-Shenzhen, according to the manufacturer’s instructions (Illumina). For each medium, the 40-hour and 72-hour samples were mixed equally and one 200-bp paired-end library was prepared and sequenced.

### Mapping of RNA-Seq reads

The RNA-Seq reads were mapped to the *Tl* genome [GenBank: ANBJ01000000], using TopHat version 1.1.4 [26]. TopHat uses the ultrafast short read mapping program bowtie [29] to align RNA-Seq reads to the genome. For short reads (<75 bp), TopHat only reports GT/AG introns, and for longer reads (≥75 bp), it also reports GC-AG and AT-AC introns. TopHat alignment was performed with default parameter settings, except that “min-intron-length” was set as 10 and “max-intron-length” as 5000.

Read alignments in the “accepted_hits.sam” file from each TopHat run were examined to retrieve uniquely mapped paired-end reads with an interval length <5 kbp. An interval length < 0 (i.e., the paired-end reads were partially overlapped with each other) was allowed if the alignments of paired-end reads did not contradict each other. In addition, the strands to which the reads were mapped were also estimated based on the donor-acceptor sequences if the reads contained introns. The paired-end reads were excluded if the paired-end reads were mapped to different strands of genome sequence. To reduce the alignment error at the ends of reads, the length of the terminal anchor had to be at least 5 bp; otherwise, the terminal anchor bases were trimmed. We also reduced alignment error by finding cases where the 3’ splice site of a candidate SJ (retrieved directly from RNA-Seq alignments) was located within 6 bp of 5’ end of a read or the 5’ splice site of a candidate SJ was located within 6 bp of 3’ end of a read. In these cases, the overlapping bases between the SJ and the read sequence were trimmed off the read if the total length of the terminal anchor was > 12 bp. The resultant alignment of RNA-Seq reads was used to construct transcript units and predict SJs.

### Transcript units

Transcriptionally active regions were predicted as a contiguous expression region longer than 35 bp with each base supported by at least four reads. The adjacent transcriptionally active regions were further grouped into a transcript unit if they were joined by at least one set of paired-end reads. Transcript units longer than 150 bp and with an average expression of ≥ 10 reads per base were analyzed in this study.

### RNA-Seq based models

To discover additional genes and obtain more reliable models, we assembled the mapped PDA + MSM reads into RNA-Seq based models (rnaseq set). First, we created artificial reads to include both the read pair bases and the interval bases. For a read pair with an interval < 0 bp, an artificial read was created by merging the paired-end reads. For a read pair with an interval ≥ 0 bp and < 5 kbp, the interval was examined to determine whether there were SJs (see section “Detection of splice junctions” for details) located in this interval. For read pairs with no SJs in the interval, all the interval bases were treated as exonic regions to create artificial reads. For read pairs with only one SJ located in the interval and with interval length - SJ length < 85 bp, the SJ was included in the artificial read. For other read pairs, no artificial reads were created. Next, we pooled together all the artificial reads and the reads that did not have a corresponding artificial read to create rnaseq models by enumerating all possible overlap among the pooled reads. Models with a length ≥ 100 bases and each base supported by at least two reads were analyzed in this study. Finally, we obtained 22,231 models by merging the overlapping models into the same loci, linking the neighboring partial models (distance ≤ 30 bp, and in the same loci) together and linking neighboring loci (distance ≤ 10 bp) together. The above procedure for assembly of reads was implemented in self-written PERL scripts.

Among the 22231 models, 14701 models overlapped with fggw models by over 10 bp and were excluded from further analyses. For the resultant 7530 models, the exonic regions were scanned to find all potential start codons and stop codons in all six possible frames (or three possible frames, depending on whether the direction can be determined based on SJ sites). All of the possible coding sequences were determined by combining the adjacent start codon and stop codon from all of the possible frames, and the longest one was obtained for each model. As a result, a total of 1119 models (including both complete models and partial models) were determined to have a coding sequence > 30 aa. Finally, 1111 rnaseq models were obtained by further excluding eight models overlapping with rRNA and tRNA. All the 1111 rnaseq models are supported by no stop codons in the coding sequences. Among them, the complete models are also supported by a start codon and a stop codon. A non-redundant (nr) set of protein models (10520) were created by combining the fggw models (9409) and the rnaseq models (1111).

### Assessment of gene transcription levels

The gene transcription levels of all nr models in media PDB and MSM were calculated and compared using the Cufflinks package version 0.9.3 with default parameter settings and were measured as FPKM, which is analogous to single-read “RPKM” [27].

### Detection of splice junctions

Candidate SJs supported by at least two reads were subjected to the following filters and corrections. First, we only included SJs with a branch site sequence located within 60 bp from the 3’ss (see below). Then, we isolated SJ pairs that overlapped and had the same length. These SJ pairs may represent alignment errors rather than AS. Here, an overlap < 10 bp was used to indicate the presence of errors in alignment. The SJ forms that had lower 5’ss scores (see below) were regarded as erroneous SJs and were removed with its count added to the correct SJ. As a result, 88 SJ pairs were subject to correction for the pooled PDB and MSM reads. Finally, we excluded one SJ because of the presence of an ‘N’ in the intron sequence for the convenience of splice score calculations. The obtained SJ set, named TopHat_Rev set, was used to analyze the splice site sequences and alternative splicing.

Sequence logos were created using WebLogo 3 (http://weblogo.threeplusone.com/) [30].

### Identification of alternative splicing events

The TopHat_Rev set of SJs were clustered based on overlap and were evaluated for AS. The clusters containing SJs both on plus strand and on minus strand were excluded.

A cluster of two SJs were classified as A5SS if the two SJs had common 3’ss. Similarly, a cluster of two SJs were classified as A3SS if the two SJs had common 5’ss. Following Marquez et al. [6], a cluster of two SJs were classified as “A5SS and A3SS” if one SJ had a smaller 5’ss coordinate and a bigger 3’ss coordinate than the other and were classified as “A5SS or A3SS” if one SJ had both a smaller 5’ss coordinate and a smaller 3’ss coordinate than the other. A cluster of three or more SJs were classified as MA5SS if all the SJs shared common 3’ss and were classified as MA3SS if all the SJs shared common 5’ss.

Exon-skipping was defined as a cluster of three SJs in which the first SJ and the second SJ did not have common bases, while the third SJ shared common 5’ss with the first SJ and shared common 3’ss with the second. Using this criterion, no exon-skipping events were observed in the clusters with at least three SJs. Other clusters were encoded by a combination of the total number of SJs and the numbers of A5SS, A3SS, “A5SS and A3SS”, and “A5SS or A3SS” identified by pairwise comparisons of all SJs in the cluster.

The clusters containing only one SJ were subject to IR analysis. The filtered RNA-Seq reads were used to calculate coverage for each base of genome. When an intron had sites with coverage ≥1, it was classified as a retained intron otherwise it was classified as a constitutive intron. The intron retention ratio was calculated for IR, using the median coverage of intronic sites divided by the reads supporting the SJ [6].

### Branch sites and polypyrimidine tracts

The branch site and polypyrimidine tract for each intron were identified using a motif searching method implemented in a self-written PERL script, as described in previous studies [28,31].

The sequence CURAY was used as the primary motif, UUARY was used as the secondary motif, and a modified YURAY motif in which either the first, third, or fifth position was allowed to be any nucleotide was used as the alternate motif. The PERL script reported the 3’-most instance of the primary motif, if exists, as the branch site; otherwise, it reported the 3’-most instance of the secondary motif as the branch site. If there was no observed instance of the primary motif or secondary motif, the alternate motif was used and the 3’-most instance, if exists, was reported as the branch site.

The polypyrimidine tract was defined as at least six consecutive nonadenine nucleotides containing no fewer than three uridines [32-35]. For simplicity, only the 3’-most six consecutive nonadenine nucleotides were reported in this study. Instances containing a maximal number of Ts were searched and if more than one instance was observed for each intron, the 3’-most one was reported as the polypyrimidine tract.

### Position weight matrices and splice site scores

Position weight matrices and splice site scores were calculated following Sheth, et al. [36]. Sequences of 5’ss, 3’ss, and branch site of SJs were separately aligned using the intron-exon junction or branch point A as the anchor. For 5’ss, the length was 13 bases (including 10 intronic bases and 3 exonic bases). For 3’ss, the length was 17 bases (including 14 intronic bases and 3 exonic bases). For the branch site, the length was 11 bases (including the branch point A, 6 bases upstream, and 4 bases downstream). The alignments were used to calculate position weight matrices which represented the nucleotide frequencies at each position. Each position weight matrix had four rows (for A, T, G, and C, respectively), and the number of columns was equal to the length of alignment. A log-odds matrix was constructed from the position weight matrix by using the base 2 logarithm of the ratio of frequencies at each matrix position against a background frequency of 0.25 for each nucleotide.

The 5’ss, 3’ss, and branch site sequences of each SJ were scored based on the corresponding log-odds matrix by adding up the entities in the matrix that corresponded to the nucleotides at each position on the sequence. In this study, all the SJs were first used to calculate the matrix, and then, the matrix was used to score each SJ. Compared with only using a small subset of SJs, e.g., IC, a matrix based on all of the SJs avoided a logarithm of zero and therefore did not need to introduce a pseudocount that may bias the distribution of scores. In addition, different from previous studies using position weight matrices [8,33], we used raw scores rather than rescaled scores because separately rescaling the positive scores and the negative scores may also affect the distributions of scores, particularly for cases with both positive scores and negative scores.

### Validation of AS by RT-PCR

A total of 45 IR, three A5SS, and five A3SS events were randomly selected and validated by RT-PCR. *Tl* was cultured in PDB, and mycelia were collected at 48 hours. RNA was extracted using a Fungal RNA Kit (OMEGA), and RT-PCR was performed using a PrimeScript®RT reagent Kit with a gDNA Eraser (Perfect Real Time) (TaKaRa) following the manufactures’ instructions. Two pairs of exclusive primers were designed for each selected AS event. For IR, primers in the intron were designed to indicate the retained isoform and primers in exons were designed to indicate the spliced isoform. For A5SS and A3SS, primers were designed to span the splice junction to distinguish different isoforms. For each isoform, 35 cycles of PCR were performed, and the PCR products were verified by sequencing.

### Availability of supporting data

The Illumina reads have been deposited in the Sequence Read Archive at NCBI (http://www.ncbi.nlm.nih.gov/sra) and they are available under study accession number SRP040673.

## Additional files

Additional file 1:
**Contains seven tables (Tables S1-S7) listing information on sequencing, splicing junction, and alternative splicing.** **Table S1.** lists annotations for new gene models predicted based on RNA-Seq. **Table S2.** lists annotations and transcription levels for all gene models. **Table S3.** lists detailed information for all splice junctions (SJs) based on PDB + MSM RNA-Seq data (TopHat_Rev). **Table S4.** lists detailed information for all groups of splice junctions (SJs). **Table S5.** lists detailed information for all primers designed for validation of alternative splicing (AS) events. **Table S6.** lists mean, standard deviation, and median values of lengths, GC contents, 5'ss scores, 3'ss scores, and branch site scores of introns. **Table S7.** lists Mann-Whitney-Wilcoxon tests of lengths, GC contents, 5'ss scores, 3'ss scores, and branch site scores of introns. Additional file 2:
**Contains four figures (Figures S1-S4) listing the RT-PCR validation of alternative splicing events, sequence logos of the splice sites and features of retained introns (IR).** **Figure S1.** Validation of intron retention (IR) with RT-PCR. **Figure S2.** Validation of alternative 5’ splice site (A5SS) (A) and alternative 3’ splice site (A3SS) (B) with RT-PCR. **Figure S3.** Sequence logos of 5’ splice site (left), branch site (middle), and 3’ splice site (right) sequences of constitutive introns (IC), retained introns (IR) of different intron retention ratio bins, introns of alternative 5’ splice site (A5SS), and introns of alternative 3’ splice site (A3SS). **Figure S4.** Features of retained introns (IR). “High coverage” refers to those supported by at least two independent reads at all positions of the retained intron and “low coverage” refers to the other retained introns. Both IR (high coverage) and IR (low coverage) were binned based on intron retention ratio (IRR).

## Abbreviations

A3SS: Alternative 3’ splice site
A5SS: Alternative 5’ splice site
AS: Alternative splicing
FPKM: Numbers of fragments per kilobase of exon per million fragments mapped
IA-IR: Alternatively spliced introns except retained introns
IC: Constitutive introns
IR: Intron retention
IRR: Intron retention ratio
MA3SS: Multiple-alternative 3’ splice site
MA5SS: Multiple-alternative 5’ splice site
MSM: Modified mineral salt medium
PDB: Potato Dextrose Broth
RNA-Seq: Transcriptome sequencing using high-throughput sequencing technology
SJ: Splice junction
*Tl*: *Trichoderma longibrachiatum*
5’ss: 5’ splice site
3’ss: 3’ splice site
