# Supplementary material for: Deep RNA sequencing reveals a high frequency of alternative splicing events in the fungus Trichoderma longibrachiatum
Source: BMC Genomics. 2015 Feb 6;16(1):54. doi: 10.1186/s12864-015-1251-8 (PMC4324775; doi:10.1186/s12864-015-1251-8)
Supplement: Additional file 2: — Contains four figures (Figures S1-S4) listing the RT-PCR validation of alternative splicing events, sequence logos of the splice sites and features of retained introns (IR). Figure S1. Validation of intron retention (IR) with RT-PCR. Figure S2. Validation of alternative 5’ splice site (A5SS) (A) and alternative 3’ splice site (A3SS) (B) with RT-PCR. Figure S3. Sequence logos of 5’ splice site (left), branch site (middle), and 3’ splice site (right) sequences of constitutive introns (IC), retained introns (IR) of different intron retention ratio bins, introns of alternative 5’ splice site (A5SS), and introns of alternative 3’ splice site (A3SS). Figure S4. Features of retained introns (IR). “High coverage” refers to those supported by at least two independent reads at all positions of the retained intron and “low coverage” refers to the other retained introns. Both IR (high coverage) and IR (low coverage) were binned based on intron retention ratio (IRR). [file 12864_2015_1251_MOESM2_ESM.pdf]

Deep RNA sequencing reveals a high frequency of alternative splicing events in the fungus *Trichoderma longibrachiatum*

Supplementary Figures

Supplementary Figure Legends

Supplementary Figure S1. Validation of intron retentions (IR) using RT-PCR. For each splice junction (SJ), two exclusive primers were designed and RT-PCR was performed. The arrows indicated the bands that were verified by sequencing. "+" indicated that both spliced form and retained form were verified by sequencing.

Supplementary Figure S2. Validation of alternative 5' splice site (A5SS) (A) and alternative 3' splice site (A3SS) (B) using RT-PCR. For each alternative splicing (AS) event, two exclusive primers were designed and RT-PCR was performed. The arrows indicated the bands that were verified by sequencing. "+" indicated that both spliced form and retained form were verified by sequencing.

Supplementary Figure S3. Sequence logos of 5' splice site (left), branch site (middle), and 3' splice site (right) sequences of constitutive introns (IC), retained introns (IR) of different intron retention ratio bins, introns of alternative 5' splice site (A5SS), and introns of alternative 3' splice site (A3SS).

Supplementary Figure S4. Features of retained introns (IR). "High coverage" refers to those supported by at least two independent reads at all positions of the retained intron and "low coverage" refers to the other retained introns. Both IR (high coverage) and IR (low coverage) were binned based on intron retention ratio (IRR).

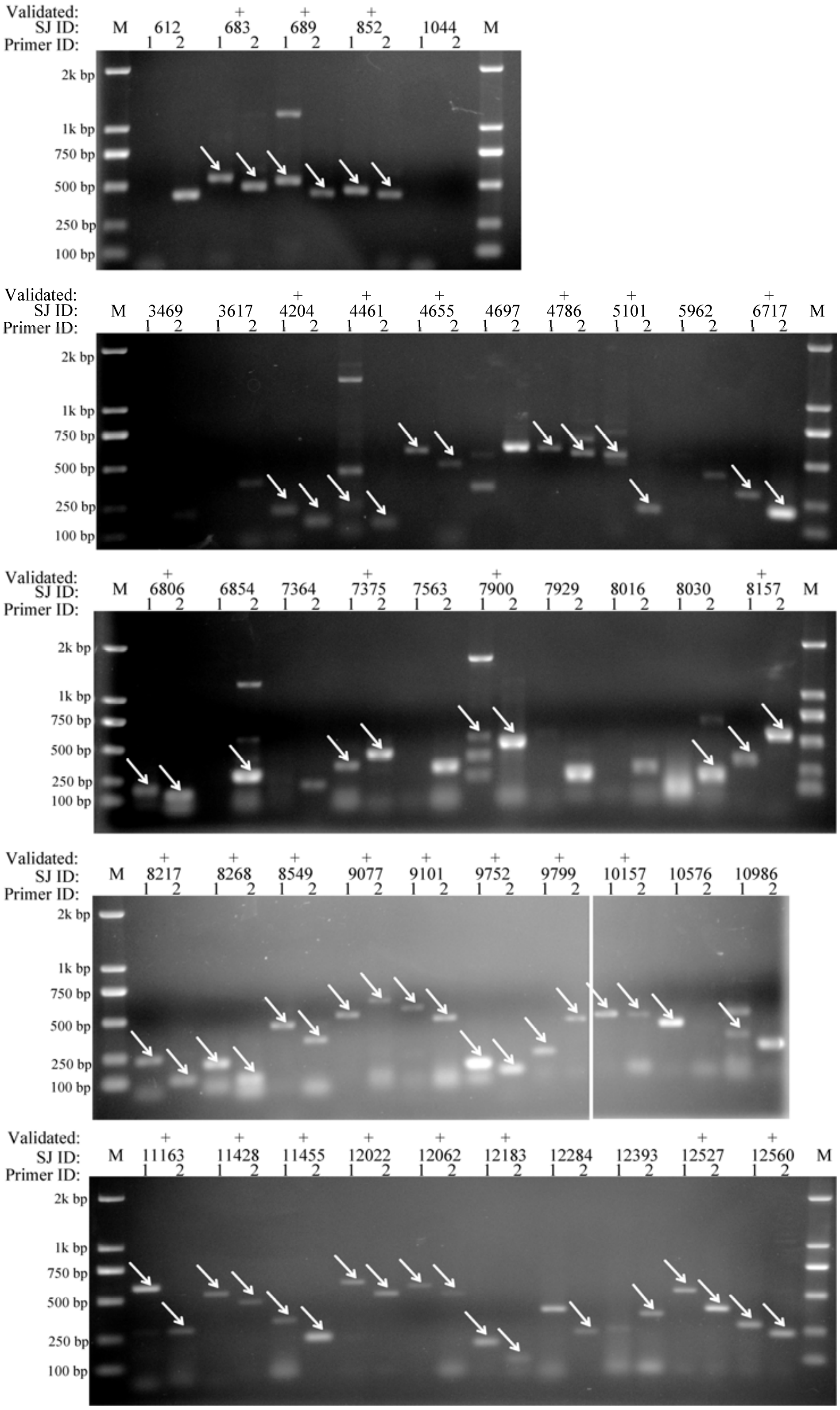

Supplementary Figure S1.

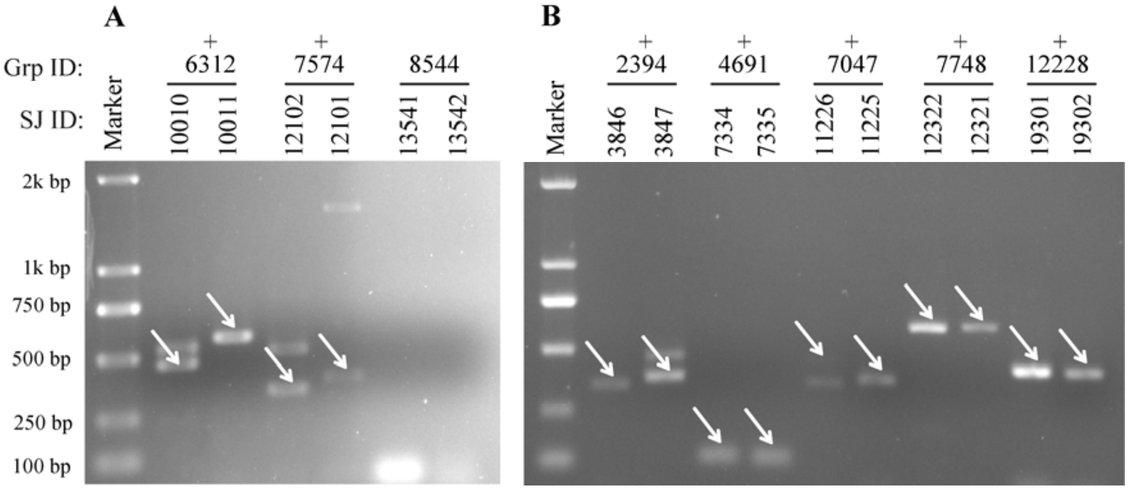

Supplementary Figure S2.

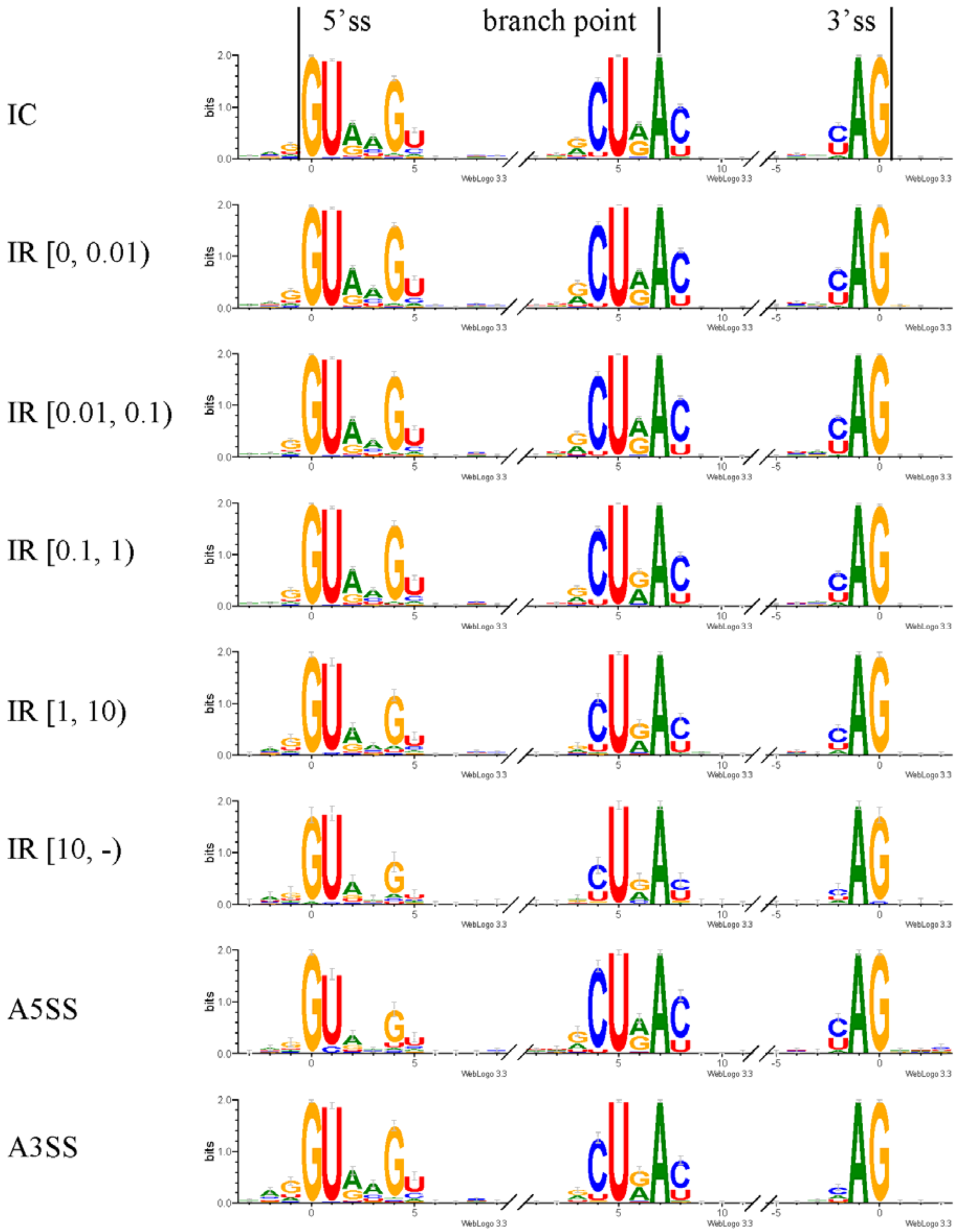

Supplementary Figure S3.

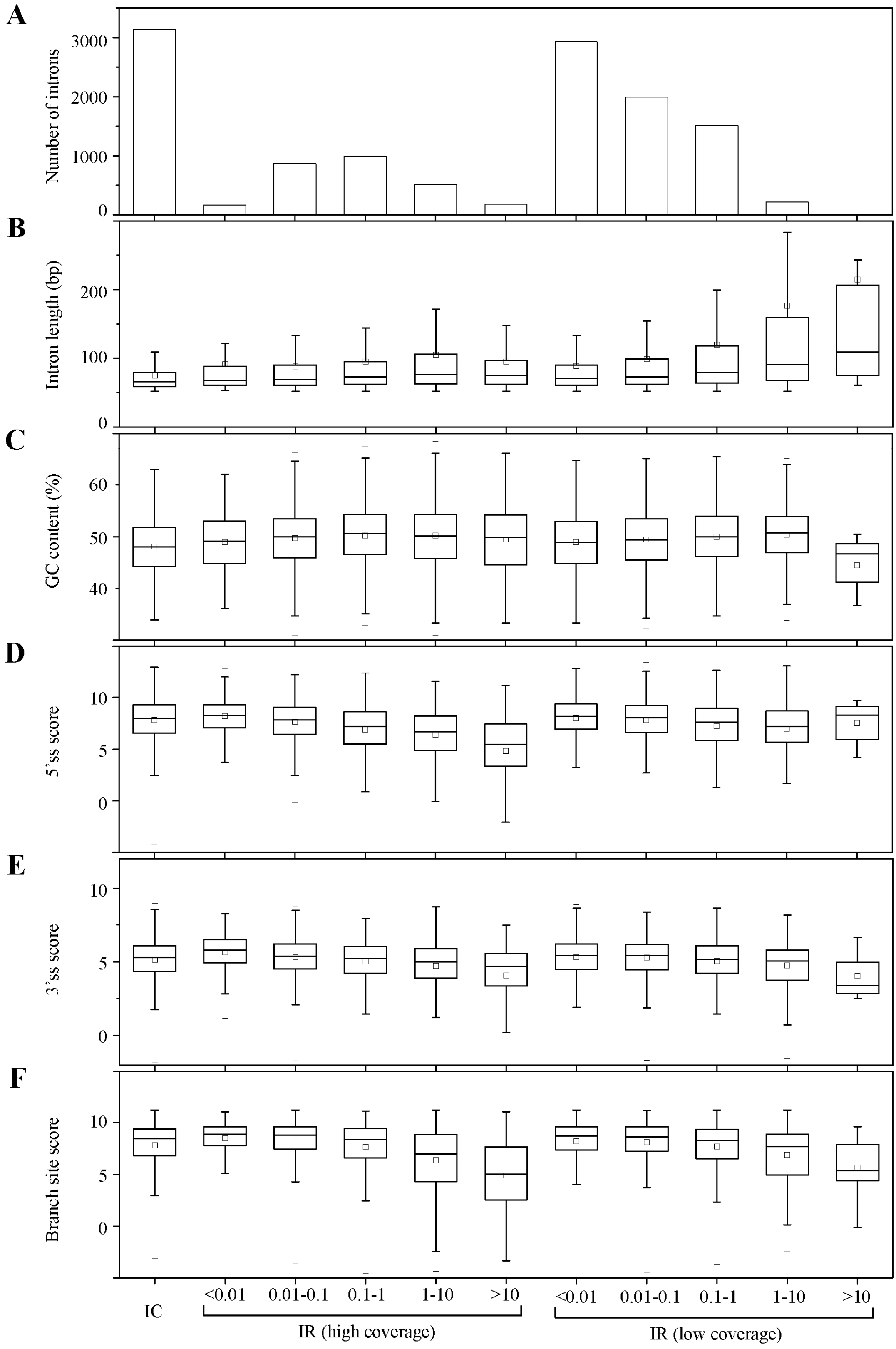

Supplementary Figure S4.
